# Prevalence of Intestinal Helminths Infestation in Children Attending Princess Marie Louise Children's Hospital in Accra, Ghana

**DOI:** 10.1155/2017/8524985

**Published:** 2017-09-05

**Authors:** Robert Mirisho, Margaret L. Neizer, Bismark Sarfo

**Affiliations:** ^1^Department of Epidemiology and Research Methodology, St. Francis University College of Health and Allied Sciences, Ifakara, Tanzania; ^2^Department of Epidemiology and Disease Control, School of Public Health, University of Ghana, Legon, Accra, Ghana; ^3^Princess Marie Louise Children's Hospital, P.O. Box GP 122, Accra, Ghana

## Abstract

The deworming exercise program does not cover all children who are not in school. This study determined the prevalence and species type of helminth infestation and associated factors among children attending Princess Marie Louise Children's Hospital in Accra, Ghana. Children (225) below the age of 10 who have not taken antihelminthic drugs prior to the study period were recruited between May and June 2015. Children or guardians were interviewed using structured questionnaires and fresh stools were collected and processed for helminths species identification using microscopy. Data were analyzed using Stata version 12. Overall helminths infestation prevalence was 17.33% (39/225). The identified species were hookworm (10.22% (23/225)) and* Ascaris lumbricoides* (7.11% (16/225)). No double infestation was observed. Significant associations were observed between infestation and age group beyond 4 years (48 months) (aOR = 16.72, 95% CI 1.00–279.72), place of residence (aOR = 7.35, 95% CI 1.68–32.11), washing hands after using toilet (0.04, 95% CI 0.01–0.20), and dirt on fingernails of children (7.96, 95% CI 1.73–36.65). This study demonstrates high prevalence of helminths parasites, hookworm, and* Ascaris lumbricoides* in children attending PMLCH. Deworming exercise should be extended to children hospitals in developing countries.

## 1. Background

Historically, soil-transmitted helminths (STH) infestations were prevalent in developed countries but sustained control efforts and economic development helped to eliminate it. In many parts of sub-Saharan Africa and Southeast Asia, there had, until recently, been little change in the prevalence of STH over the last half of the 20th century.

In the last ten years, however, there has been increased political and financial support for the global control of STH infection, with a strong focus on school-based deworming. Where scaling-up of treatment has happened it has occurred in changing social and economic contexts, including increased urbanization. Such a changing landscape necessitates accurate description of the contemporary distribution of STH transmission and populations at risk, and this information can inform the estimation of the global burden of STH [1].

The common STH parasites include* Ascaris lumbricoides (A*.* lumbricoides)*, hookworm, and* Trichuris trichiura (T. trichiura)* and they cause human infection through contact with their eggs or larvae that thrive in the warm and moist soils, especially in the tropics.

World Health Organization (WHO) report indicates that about 1221–1472 million cases of ascariasis, 750–1050 million cases of trichuriasis, and 740–1300 million cases of hookworm infestation are reported annually, globally [2].

*A*.* lumbricoides* causes ascariasis which is widespread due to a variety of medical and surgical complications (Raghu et al., 2012). It has been estimated that 1.5 billion cases of infection globally and 65,000 deaths occur due to* A*.* lumbricoides *[3].

Hookworm is one of the most common chronic infections with an estimated 1.3 billion cases globally and directly accountable for 65,000 deaths annually [4]. Clinical manifestations of hookworm disease are the consequences of chronic intestinal blood loss and iron-deficiency anemia (Anah et al., 2008).

*T. trichiura,* which is like* A*.* lumbricoides *and hookworm, affects the physical and mental development in children. About 1.1 billion cases of infection and 70,000 deaths occur due to* T. trichiura* annually [4].

There could be single or multiple infestation of these parasites. Children of school age and immune-deficient individuals are particularly vulnerable to these parasitic helminth infestation, with heavy infections associated with cognitive impairment, iron-deficiency anemia, growth retardation, malabsorption, and malnourishment [5]. These parasites also cause different levels of tissue damage and ill-health to human and they are major health threat to the development of children throughout the world, especially in developing countries. Currently, there is a deworming exercise on-going in Ghana under the auspices of the Ministry of Health but this exercise is only for children of school-going age. Hence occupational risk groups and non-school-going children cannot benefit from this program.

Moreover, studies assessing the prevalence of helminths infestation among children attending children hospitals for different illnesses are important in providing better understanding in guiding policies in the deworming exercise. Despite the increasing interest in helminth infestation causing anemia and retarded growth in children, only few studies have assessed the intestinal helminth infestation in children and its risk factors in Ghana. The prevalence of intestinal helminths infestation and its risk factors in children attending children hospitals for different illnesses in Ghana have not been previously reported. This study determined the prevalence of intestinal helminth infestation among children attending Princess Marie Louise Children's Hospital in the Greater Accra Region of Ghana.

## 2. Methods

### 2.1. Study Design

A cross-sectional study design was used to conduct this study using interview questionnaire and microscopy to determine helminths infestation and associated factors in children.

### 2.2. Study Setting

The study was carried out at Princess Marie Louise Children's Hospital (PMLCH). Princess Marie Louise Children's Hospital is a healthcare institution located in Accra, the capital city of Ghana, and is the largest hospital for treating children with severe malnutrition in Ghana. It receives referrals for rehabilitating children with nutritional disorders from hospitals in Accra and occasionally from other regions. It is a 74-bed children's hospital situated in the commercial centre of the capital city, Accra. It has over 70,000 visits from children presenting with a variety of conditions to its outpatients department in 2012. Thus, it attends to patients brought in by their parents/guardian (self-referred) as well as children referred from other healthcare facilities in Accra.

Study involving 225 children was conducted between May 2015 and June 2015.

### 2.3. Sample Size

The capacity of the laboratory to accommodate the daily routine examination of the stool samples informed the sample size estimation. Based on a similar cross-sectional study conducted in Ethiopia by Abera and Nibret [6], which identified the prevalence of intestinal helminths to be 45% in the study area, the sample size for the current study was determined to be 194 and, with a nonresponse rate of 10%, the final sample size for the study was estimated at 225.

### 2.4. Participants Recruitment

The study population was children attending the Princess Marie Louise Children's Hospital between May and June 2015 in the Greater Accra Region of Ghana for illnesses such as malaria, diarrhea (cholera), and fever. Participants' recruitment was done using consecutive sampling after consent and assent were obtained. Children up to 10 years attending the hospital for illnesses as described above who have not taken any antihelminthic medication two weeks prior to the study were enrolled. 

### 2.5. Data Collection

A closed-ended interview questionnaire was administered to 250 children or their parents. Each child or parent was given a unique identification. Questions, including educational level, parents' occupation, availability of toilet facility at home, a habit of using the toilet, and hand washing after using toilet, were answered by participants. Subsequently, finger nails of children were observed to determine if they were cleaned or had dirt.

Stool sample collection vials labelled with the unique identification of the participants and toilet tissue papers were provided to children and guardians, and instructions were provided on how to collect and bring the stool samples. After collecting sufficient fresh stool sample in plastic vial from each participant, the sample was taken immediately to the laboratory for examination. All the freshly collected samples were examined on the same day throughout the study period. Out of the initial 250 participants who completed the questionnaire, 25 did not provide stool samples.

In the laboratory, a drop of saline (0.85%) was placed on a clean slide; then a small piece of stool was placed on the slide and mixed and covered with a cover slip. For those samples with mucus, the examination was done without saline. The mucus is put on the slide and covered with cover slip. The processed samples were examined under 10x and 40x light microscope for identification of helminth eggs and species type. The helminths eggs identification was done using the helminths key identification by Melvin and Brooke [7] and Ash and Orihel [8]. Every tenth negative specimen and every specimen where helminth eggs were identified were also confirmed by a senior laboratory technician. Presence or absence of parasites in stools was coded and entered into Microsoft Excel program 2010 version every day and subsequently imported into Stata 12 for statistical software.

This study received ethical approval from the Ghana Health Service Ethics Review Committee (GHS-ERC: 34/02/15). Also, permission was obtained from the Head of Princess Marie Louise Children's Hospital before commencement of the study. Finally, parents/guardians completed an informed consent and assent forms before the study started. Parents/guardians were communicated to about the outcome of the stool and the prescription of mebendazole by the resident physician was made to those who tested positive.

### 2.6. Statistical Analysis

Data was analysed using STATA version 12 statistical software. Chi-square was used to test associations between independent variables and helminths infestation (main outcome). Univariate and multivariate logistic regression analysis were performed with odds ratio (OR) and 95% confidence interval (CI) to determine the association between the helminths infestations and associated factors. *P* < 0.05 was considered to be statistically significant.

## 3. Results

### 3.1. Demographic Characteristics and Overall Prevalence of Intestinal Helminth Infestation in Children

A total of 250 participants (127 males and 123 females) aged 0–10 years or 0–120 months participated in the study. The mean age of the participants was about 4 years or 47.56 months. Of the total 225 stool samples examined from 225 participants, the overall prevalence of intestinal helminths was 17.3% (39/225).

Among these, a total of 64.1% (25/39) were males and 35.9% (14/39) were females and they were all positive for only single infection. No double infestation was detected and there was no significant difference in helminths infestation between males and females (Table 1).

Hookworm was the predominant intestinal helminth species with a prevalence of 10.2% (23/225) followed by* A*.* lumbricoides *(7.1%; 16/225) (Table 2).* T. trichiura *was not detected in participants stool samples.

According to their residence, 84.9% (191/225) were from urban and 15.1% (34/225) children were from rural areas. 11.0% (21/191) of the children from the urban and 52.9% (18/34) from the rural areas were infested with intestinal helminths and this difference in infestation between urban and rural children was statistically significant (*P* < 0.05) (Table 1).

Among the age groups, the highest prevalence was 43.3% (29/67) for those between 72 and 120 months, 13.3% (4/30) for those between 48 and 71 months, and 9.3% (5/54) for those between 24 and 47 months and the least was 1.4% (1/74) for children between 0 and 23 months and the difference of infestation between these age groups was statistically significant (*P* < 0.05) (Table 1).

According to their parents' occupation, 76.4% (172/225) were employed and 23.6% (53/225) of parents were not employed. 9.9% (17/172) of children whose parents were employed and 41.5% (22/53) of children whose parents were not employed were positive for intestinal helminths (*P* < 0.05) (Table 1).

According to parents' education, 12.4% (28/225) have never been to school, 4.9% (11/225) had primary level education, and 68.00% (153/225) had secondary school level education. 14.7% (33/225) had tertiary level education and this difference between parents educational level is statistically significant (*P* < 0.05).

For place of convenience, 80.0% (180/225) have latrine at home and 20.0% (45/225) do not have latrine at home. 8.9% (16/180) of those who have latrine at home and 51.1% (23/45) of those who do not have latrine at home were infected with intestinal helminths and the difference between these groups was statistically significant (*P* < 0.05) (Table 1).

Among 225 children who participated in this study 80.9% (182/225) had indicated washing hands after using toilet and 19.1% (43/225) reported not washing hands after using the toilet. 5.5% (10/182) who washed hands after using the toilet and 67.4% (29/43) who did not wash hands after using the toilet were positive for helminths infestation and the difference was significant (*P* < 0.05) (Table 1).

Visually observing the fingernails of the participants revealed that 45.8% (103/225) of the children had dirt in their fingernails at the time of visiting the hospital. 33.9% (35/103) with dirt in their fingernails and 4/122 (3.3%) without dirt in their fingernail were positive for intestinal helminths and the difference was significant (*P* < 0.05) (Table 1).

### 3.2. Prevalence of Intestinal Helminths Species by Sex of Participants

Comparing the differences in infestation between males and females, 8.7% (11/127) of males and 6.1% (6/98) of females were infested with* Ascaris lumbricoides *while 11.8% (15/123) of males and 8.2% (8/98) of females were positive for hookworm although the difference was not statistically significant (Table 2).

### 3.3. Factors Associated with Helminths Infestation in Study Participants

In the univariate logistic regression model, age group beyond 47 months, residence (cOR = 10.25, 95% CI 4.54–23.16), occupation (cOR = 7.0, 95% CI 3.34–14.63), parents education and those with secondary education and above, latrine at home (cOR = 0.09, 95% CI 0.04–0.19), those who wash hands after using toilet (cOR = 0.03, 95% CI 0.01–0.06), and those having dirt in finger nail (cOR = 15.85, 95% CI 5.41–46.47) were significantly associated with increased risk of helminths infestation (*P* < 0.05) (Table 3).

Adjusting for significant variables in the univariate analysis, multivariate analyses showed that age groups, 48–71 months (aOR = 16.72 (1.00–279.72)), 72–120 months (aOR = 51.87 (3.87–694.71)), residence (aOR = 7.35, 95% CI 1.68–32.11), hand washing after using toilet (aOR = 0.04, 95% CI 0.01–0.20), and dirty material in fingernails (aOR = 7.96, 95% CI 1.73–36.65) were significantly associated with helminths infestation (Table 3).

Children aged 48–71 months were 16.72 (aOR = 16.72 (1.00–279.72)), more likely in getting helminths infestation compared with those below 24 months (Table 3). Also children aged 72–120 months were 51.87 (aOR = 51.87 (3.87–694.71)), more likely contracting helminths infestation than those below 24 months (Table 3).

Children from rural area were 7.35 (aOR = 7.35, 95% CI 1.68–32.11) more likely to be infested with gastrointestinal helminths than those from urban areas (Table 3).

Meanwhile, children who wash their hands after using toilet were 0.04 less likely to be infested than those who did not wash their hands (aOR = 0.04, 95% CI 0.01–0.20) (Table 3).

Similarly, the odds of being infested with gastrointestinal helminths in those children whose fingernails contained dirty material were 7.96 more likely than those who did not have dirty materials in their fingernail (aOR = 7.96, 95% CI 1.73–36.65) (Table 3).

## 4. Discussion

In this study which forms part of the thesis submitted by Mishiro to the University of Ghana in 2015, the overall prevalence of intestinal helminth infestation was 17.33%. It was lower when compared to other studies which were done among children in different countries. Prevalence of 41.46% in a district in Northern Ethiopia [6] and 31% from rural Kenya [9] was reported. Also a prevalence of 40.5% and 40.7% among preschool age children and school age children, respectively, was reported in Kenya [10]. However, the prevalence of helminth infestation in the current study was relatively higher than other studies conducted in children in the Ashanti region (11.1%) of Ghana [11] and in Osun State in Nigeria (12.2%) [12]. The difference in prevalence could be attributed to timing of the study, sampling of study participants, seasonal differences in conducting the study, environmental conditions, and other geographical factors in these study areas. Nonetheless, the prevalence of the parasitic infestation in all the studies has all been appreciably high.

Helminths infestation in children as reported in this study has been associated with 48–71 months' age group, lack of hand washing habit after using toilet, presence of dirty material in fingernails, and residence location of the participants, either urban or rural.

In terms of the age group infected with the parasites, the result of the present study is comparable to previous studies conducted among children in Kumasi in Ghana which revealed that the proportion of helminth infestations among children aged less than one year (12 months) and between 1 and 9 years (12 months to 108 months) was 1.3% and 10.8%, respectively [13].

Although the study did not capture data on children who are currently in school, with the Free Compulsory Universal Basic Education (FCUBE) policy in Ghana, children 4 years and older must be in school, and indeed a study conducted in Kintampo North in the Brong Ahafo Region of Ghana indicated that 74% of children aged 5–15 years were enrolled in primary school and the prevalence of hookworm in the municipality has been reported as 45% [14].

In the current study two common intestinal helminths species were identified in the stool samples of the participants, the most common one being hookworm, followed by* A*.* lumbricoides* with prevalence of 10.22% and 7.11%, respectively. Similar results were reported from Tilli town in Ethiopia [15] with hookworm prevalence of 7.8%.

Interestingly, boys had a higher prevalence of helminths infestation than girls in the current study although the difference is not statistically significant. Previous study had also reported that there was no statistical significant difference between the prevalence of intestinal infestation across gender [16]. Both genders could be equally exposed to factors such as poor hand washing and presence of toilet facilities at home among other factors associated with helminths infestation.

This study demonstrated that there was significant association between hand washing after using toilet and rate of intestinal helminth infestations. Children who reported washing their hands after using toilet were 0.04 times less likely to be infected with helminth parasites (aOR = 0.04, 95% CI 0.01–0.20) compared with children who do not wash their hands after using the toilet. A recent cluster randomized control trial study showed that handwashing with soap significantly reduces intestinal parasite infection in children [17]. This study further revealed that handwashing significantly decreased the prevalence of anemia in children [17]. Indeed poor handwashing has been associated with high intestinal parasite infection in Ethiopia [15].

Also there was a significant association between helminth infestation and dirty material in children fingernails. Children with fingernails containing dirty materials were 7.96 times more likely to be infected with helminths parasites compared with those without dirty materials in their fingernails (aOR = 7.96, 95% CI 1.73–36.65). This is due to poor hygienic practice, socioeconomic status, and also playing habit of children with soil. A study by Abdulkader et al. [17] in Ethiopia showed that weekly nail clipping of children significantly reduced intestinal parasites infection.

The current study further demonstrated that children who visited the hospital from rural areas were 7.35 times more likely to be infected with helminths than those from urban areas (aOR = 7.35, 95% CI 1.68–32.11). This finding corroborates the results of a study from Iran which reported high prevalence of intestinal parasite infection among children in rural areas than those in urban areas [18]. It is also similar to a study which showed that the prevalence of intestinal parasites was significantly higher in the rural areas than in the urban areas [19]. This is not surprising as a result of low socioeconomic conditions associated with poor hygienic conditions, lack of potable water, and water flushing toilets in the rural communities as compared with the urban centers. Although all the study participants live in the Accra Metropolis (urban), there are some disparities in terms of socioeconomic amenities in neighborhoods where they live. Such disparities in cities in Ghana are very pronounced, and this could have manifested in the high prevalence of the intestinal parasites in children from neighborhoods with poor social amenities such as potable water and toilets.

Although other studies have associated low educational level of parents to high prevalence of intestinal parasites in children [18] the data from our study do not support this assertion.

The higher prevalence of hookworm in this study correlates with behavioural, social, and economic factors that mediate potential exposure, including lack of access to latrine and overall socioeconomic status of the study participants.

## 5. Study Limitation

This is a cross-sectional study and the prevalence of helminth infestation cannot be generalized to the entire children in Ghana. Also, due to the limited sample size, the confidence intervals of some of the odds ratios appeared wide. Notwithstanding these limitations, the findings of this study corroborated that from other studies and added to the general knowledge of helminths infestations in developing countries which will help in developing strategies for the deworming exercise.

## 6. Conclusion

This study has revealed a relatively high prevalence of intestinal helminth infestations among children attending children hospital. Two species, hookworm and* A*.* lumbricoide*s, are the dominant intestinal parasites identified in this study with hookworm being the most prevalent species. Intestinal parasitic infection among the children is significantly associated with age group beyond 47 months, place of residence, washing hands after using toilet, and dirt on the fingernails of children.

## Figures and Tables

**Table 1:** Demographic characteristics and overall prevalence of intestinal helminth infestation in children.

Characteristics	Number of participants (%) *N* = 225	Positive (%) *n* = 39	Negative (%) *n* = 186	*P* value
Sex				
Male	127 (56.4)	25 (64.1)	102 (80.3)	0.28
Female	98 (43.6)	14 (35.9)	84 (85.7)	
Age (months)				
0–23	74 (32.9)	1 (1.4)	73 (98.6)	<0.0001
24–47	54 (24.0)	5 (9.3)	49 (90.7)	
48–71	30 (13.3)	4 (13.3)	26 (86.7)	
72–120	67 (29.8)	29 (43.3)	38 (56.7)	
Residence				
Urban	191 (84.9)	21 (10.9)	170 (89.1)	<0.0001
Rural	34 (15.1)	18 (52.9)	16 (47.1)	
Parent occupation				
Employed	172 (76.4)	17 (9.9)	155 (90.1)	<0.0001
Not employed	53 (23.6)	22 (41.5)	31 (58.5)	
Parents Education				
Never been to school	28 (12.4)	14 (50)	14 (50)	<0.0001
Primary school	11 (4.9)	7 (63.6)	4 (36.4)	
Secondary school	153 (68)	17 (11.1)	136 (88.9)	
Tertiary education	33 (14.7)	1 (3.1)	32 96.9)	
Latrine at home				
Has latrine	180 (80)	16 (8.9)	164 (91.1)	<0.0001
Has no latrine	45 (20)	23 (51.1)	22 (48.9)	
Hand wash after toilet				
Wash hand after toilet	182 (80.9)	10 (5.5)	172 (94.5)	<0.0001
Not washing hands	43 (19.1)	29 (67.4)	14 (32.6)	
Fingernail dirty				
Present dirt in fingernail	103 (45.8)	35 (33.9)	68 (66.1)	<0.0001
No dirt in fingernail	122 (54.2)	4 (3.3)	118 (96.7)	

**Table 2:** Prevalence of intestinal helminths species by sex of participants.

Sex	*Ascaris lumbricoides* (%)	Hookworm (%)	*P* value
Male (*n* = 127)	8.7	11.8	0.48
Female (*n* = 98)	6.1	8.2	

The overall prevalence of *Ascaris lumbricoides* is 7.11% and hookworm is 10.22%.

**Table 3:** Factors associated with helminths infestation in study participants.

Variable	Crude OR (95% CI)	*P* value	Adjusted OR (95% CI)	*P* value
Age group				
<24	Ref		Ref	
24–47	7.45 (0.84–65.72)	0.07	9.15 (0.58–144.98)	0.12
48–71	15.1 (1.74–131.00)	0.01	16.72 (1.00–279.72)	0.05
72–120	60.11 (7.85–460.38)	0.00	51.87 (3.87–694.71)	0.00
Residence				
Urban	Ref		Ref	
Rural	10.25 (4.54–23.16)	0.00	7.35 (1.68–32.11)	0.01
Parent occupation				
Employed	Ref		Ref	
Not employed	7.0 (3.34–14.63)	0.00	2.92 (0.38–22.27)	0.30
Parents education				
Never	Ref		Ref	
Primary	2.67 (0.58–12.19)	0.21	3.93 (0.31–49.74)	0.29
Secondary	0.13 (0.05–0.31)	0.00	2.4 (0.27–21.38)	0.44
Certificate & above	0.03 (0.004–0.26)	0.00	1.82 (0.08–43.55)	0.71
Latrine at home				
No	Ref		Ref	
Yes	0.09 (0.04–0.19)	0.00	0.80 (0.14–4.46)	0.79
Hand wash after toilet				
No	Ref		Ref	
Yes	0.03 (0.01–0.06)	0.00	0.04 (0.01–0.20)	0.00
Fingernail dirty				
No	Ref		Ref	
Yes	15.85 (5.41–46.47)	0.00	7.96 (1.73–36.65)	0.01

## Additional Points

*Recommendation.* It is recommended that deworming exercise should be incorporated at the outpatient department of the children hospital, and similar children hospitals could be identified in the country for this exercise, to augment the school deworming program currently on-going in Ghana. Parents who bring their children to the hospital must be educated on intestinal parasite infections.

## Data Access

The datasets for the current study are available from the corresponding author on reasonable request.
